# Hyperhomocysteinemia Associated with Multiple Organ Failure in Acute Pancreatitis Patients

**DOI:** 10.1155/2020/6960497

**Published:** 2020-01-21

**Authors:** Jiang Li, Saiqun Luo, Chaochao Tan, Ting Shi, Yupeng Wang, Hongbo Wang

**Affiliations:** ^1^Department of Clinical Laboratory, Hunan Provincial People's Hospital, The First Affiliated Hospital of Hunan Normal University, Changsha, China; ^2^School of Life Science, Central South University, 172 Tongzipo Road, Changsha, China

## Abstract

**Objective:**

This study aimed to evaluate the potential effect of hyperhomocysteinemia on multiple organ failure (MOF) in patients with acute pancreatitis (AP).

**Method:**

In this cohort study, a total of 1880 AP patients were enrolled and divided into the hyperhomocysteinemia group (study group) and the control group based on serum homocysteine (HCY) levels. Clinical data including demographics, clinical outcomes, and characteristics were collected for analysis. Risk factors of MOF in AP patients were determined by univariate and multivariate logistic regression analyses.

**Results:**

The hyperhomocysteinemia group showed higher multiple organ failure rates (31.83% vs 20.77%, *P* < 0.001), compared with the control group. A positive correlation between homocysteine level and APACHE II score was obtained by Pearson correlation analysis (*r* = 0.420, *P* < 0.001), compared with the control group. A positive correlation between homocysteine level and APACHE II score was obtained by Pearson correlation analysis (*P* < 0.001), compared with the control group. A positive correlation between homocysteine level and APACHE II score was obtained by Pearson correlation analysis (

**Conclusion:**

A high serum homocysteine level may be an independent risk factor of multiple organ failure in patients with acute pancreatitis.

## 1. Introduction

Acute pancreatitis (AP) is one of the most common gastrointestinal inflammatory diseases, and the majority of AP patients usually experienced a mild course. However, approximately 20% of AP patients could further progress into persistent organ failure and develop severe AP, resulting in high morbidity and mortality. It is still a great challenge to quickly and effectively screen out AP patients with high risk of persistent organ failure during clinical management. Previous reports have shown that many biomarkers such as triglycerides, high-density lipoprotein, and apolipoprotein A could be important predictors of persistent organ failure in AP patients [1, 2].

Homocysteine (HCY) is a sulfur-containing amino acid that has currently been recognized as an independent risk factor for cardiovascular, cerebrovascular, and peripheral vascular diseases [3, 4]. It was also reported that the level of serum HCY in patients with chronic pancreatitis was significantly higher than that of the healthy population [5], and the level of serum HCY was positively correlated with the incidence of pancreatitis [6]. In addition, hyperhomocysteinemia (high level of HCY) is considered a modifiable risk factor for stroke, possibly through an atherogenic and prothrombotic mechanism [7]. More specifically, HCY is associated with thrombosis and inflammation, and hyperhomocysteinemia may also lead to increased oxidative stress, thus resulting into the formation of inflammation.

However, the role of HCY in AP pathogenesis remains largely unknown. The aim of this study was to explore the possible relationship between multiple persistent organ failure and serum HCY levels, specially focusing on whether high serum HCY level in AP early course predisposes AP patients to development of persistent organ failure.

## 2. Materials and Methods

### 2.1. Study Design

**Figure 1:**
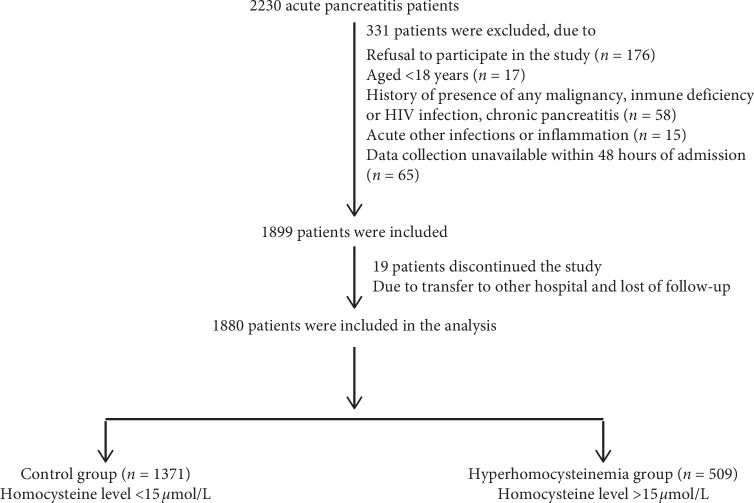
The flow chart of the study.

A cohort of 2230 AP patients was screened in the Department of emergency, Hunan Provincial People's Hospital (Changsha, China), from January 2013 to September 2017, which is part of a previously published cohort study [8, 9]. Finally, 1880 patients were prospectively enrolled in the study (Figure 1). All patients were treated as instructed by the Chinese AP Management Guidelines and followed up until discharge or hospital death. This study was approved by the Medical Ethics Committee of the Hunan Provincial People's Hospital, and written informed consent was obtained from each participant. According to the Guidelines for the Prevention of Cardiovascular Diseases in China, these AP patients were categorized into the control group (HCY < 15 *μ*mol/L) and the hyperhomocysteinemia group (HCY ≥ 15 *μ*mol/L) [10].

### 2.2. Inclusion and Exclusion Criteria

#### 2.2.1. Inclusion Criteria

(1) AP patients admitted to department of emergency, Hunan Provincial People's Hospital (Changsha, China), from January 2013 to September 2017.
(2) All the AP patients should be diagnosed with at least two of the following criteria according to revision of the Atlanta classification and definitions [11]:
  - Acute upper abdominal pain associated with AP
  - Serum amylase/lipase elevated more than three times compared with the upper limit of normal individual
  - Characteristic discovery of AP on a computed tomography scan

#### 2.2.2. Exclusion Criteria

1. Aged less than 18 years
2. Patients with a history or presence of malignancies, immune deficiency, or HIV infection
3. Chronic pancreatitis
4. Patients with other acute infection or inflammation
5. Lack of patient data within the first 48 hours of admission
6. Patients who quit halfway during the study

### 2.3. HCY Level and Data Collection

Blood samples from each participant were collected on admission within 48 h after disease onset, and the serum homocysteine levels were determined using an Automatic Biochemistry analyzer AU5821 (BECKMAN COULTER, Tokyo, Japan). All clinical data including age, sexuality, medical history, laboratory data, and clinical outcomes were collected on admission. The primary outcome used in this study was the development of persistent multiorgan failure (MOF) during admission. Secondary outcomes were pancreatic necrosis and mortality during admission.

The Acute Physiology and Chronic Health Evaluation (APACHE) scores were calculated by experienced physicians or radiologists with blinded view within the first 24 h after admission. Circulatory failure, pulmonary failure, and renal failure were defined according to the Revision of the Atlanta Classification and definitions [11]. Persistent MOF was defined by failures of ≥2 organs lasting for >48 h. Patients with a GFR of <15 were diagnosed with renal failure and treated with continuous renal replacement therapy (CRRT) [12].

### 2.4. Statistical Analysis

Approximately 20% of AP patients could further progress into persistent organ failure and develop severe AP. Assumed relative risk of hyperhomocysteinemia was 1.2; 10278 patients would be required to detect a 10% reduction of persistent organ failure for the study to attain an 90% statistical power, at a two-sided *α* of 0.05. After about first 9300 patients completed the follow-up, relative risk of the number of hyperhomocysteinemia was lower than expected, so the total sample size was increased to 17912 patients. Categorical variables were summarized in frequencies and percentages, and continuous variables were presented as the median and quartile or the mean and standard deviation, which were analyzed with the SPSS 22.0 software. The differences of categorical variables between the control and the hyperhomocysteinemia groups were analyzed through the chi-squared test. Student's *t* test or Wilcoxon test was used for analysis of continuous variables as appropriate. The correlation between the HCY level and APACHE II score was assessed by the Pearson correlation analysis. The index of *P* value < 0.1 in the univariate analysis was included in the multivariate logistic regression analysis model. Statistical significances were defined by a *P* value of < 0.05.

## 3. Results

### 3.1. Demographics Data and Clinical Characteristics of the Control and Hyperhomocysteinemia Groups

**Figure 2:**
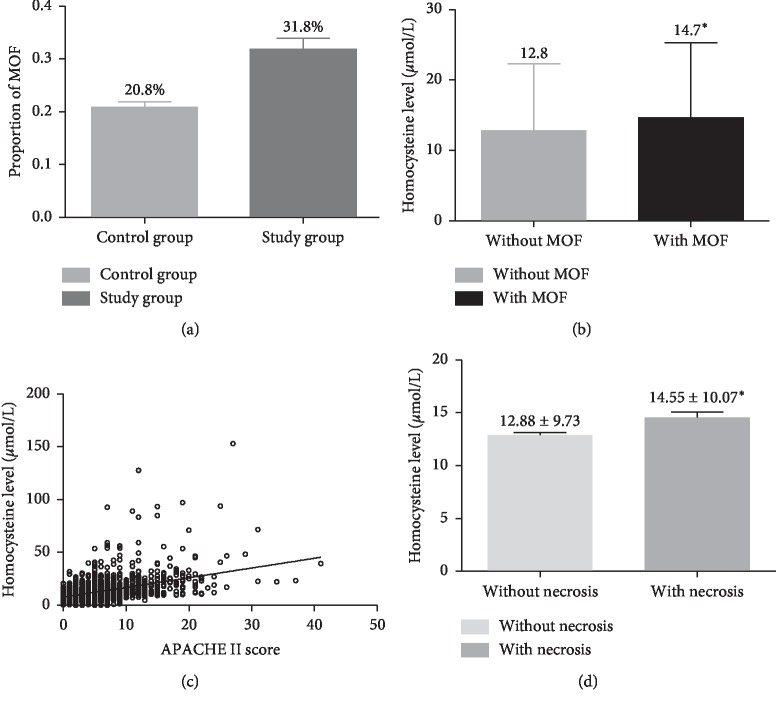
The correlation between HCYlevel and acute pancreatitis. (a) Compared with control group, *p* < 0.001. (b) Compared with patients without MOF, *p* < 0.001. (c) Pearson correlation coefficient was 0.420. (d) Compared with patients without necrosis, *p* = 0.004.

**Figure 3:**
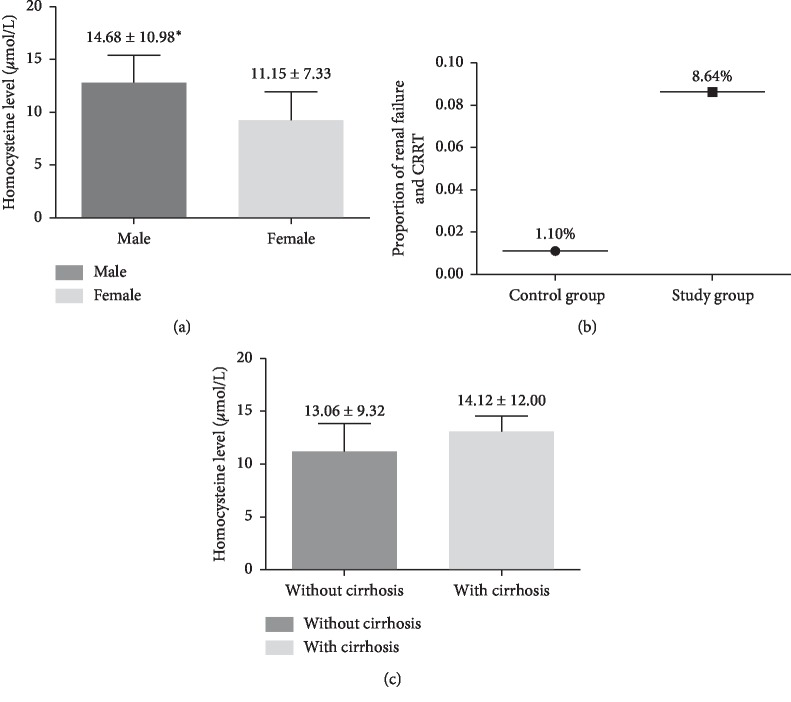
Demographics data of the control and hyperhomocysteinemia groups. (a) Female compared with male, *p* < 0.001. (b) Compared with control group, *p* < 0.001. (c) Compared with patients without cirrhosis, *p* = 0.144.

We first compared the demographic and clinical data between the control and the hyperhomocysteinemia groups (Table 1). Compared with the control group, patients of the hyperhomocysteinemia group exhibited higher incidences of biliary pancreatitis (46.76% vs 42.01%, *P* < 0.001), hypertension (21.81% vs 17.79%, *P*=0.039), diabetes (15.91% vs 9.99%, *P*=0.002), apoplexy (13.75% vs 2.33%, *P* < 0.001), multiple organ failure (31.8% vs 20.8%, *P* < 0.001) (Figure 2(a)), mortality (6.5% vs 2.0%, *P* < 0.001), and ICU admission (20.63% vs 11.45%, *P* < 0.001), and higher incidences were also observed in patients undergoing CRRT treatment (1.10% vs 8.64%, *P* < 0.001) (Table 1, Figure 3(a)). Also, patients of the hyperhomocysteinemia group were older (50.93 ± 15.58 vs 49.15 ± 13.51 years, *P*=0.015), tended to be male (73.08% vs 54.77%, *P* < 0.001), and exhibited a higher APACHE II score (8.46 ± 6.00 vs 5.13 ± 3.34, *P* < 0.001) and higher CCI (1.69 ± 1.24 vs 1.45 ± 0.98, *P*=0.023), compared with the control group (Table 1). Moreover, higher HCY levels were found in male patients compared with the females (14.68 ± 10.98 vs 11.15 ± 7.33, *P* < 0.001) (Figure 3(b)). Our analysis also showed higher pancreatic necrosis incidence in the hyperhomocysteinemia group in comparison with the control group (28.50% vs 18.70%, *P* < 0.001); AP patients with pancreatic necrosis showed higher HCY levels than patients without pancreatic necrosis (14.55 ± 10.07 vs 12.88 ± 9.73, *P*=0.004) (Table 1, Figure 2(d)), but no significant differences in AP patients with or without cirrhosis groups were observed between these two groups (14.12 ± 12.00 vs 13.06 ± 9.32, *P*=0.144) (Figure 3(c)).

### 3.2. Clinical Characteristics of Acute Pancreatitis and Severe Acute Pancreatitis

Next, we analyzed the major laboratory test values between these two groups. Compared with the non-MOF group, patients of the MOF group showed higher incidences of biliary pancreatitis (49.00% vs 41.52%, *P*=0.002), hypertension (22.82% vs 17.66%, *P*=0.030), surgery acceptance (22.15% vs 7.82%, *P* < 0.001), ICU admission (56.82% vs 0.56%, *P* < 0.001), pancreatic necrosis (84.79% vs 1.61%, *P* < 0.001), and mortality (13.65% vs 0%, *P* < 0.001) (Table 2). In contrast to the non-MOF group, patients of the MOF group showed higher levels of APACHE II score (9.81 ± 5.36 vs 4.22 ± 2.47, *P* < 0.001), CCI (2.30 ± 1.67 vs 1.37 ± 0.88, *P* < 0.001), blood urea nitrogen (8.17 ± 7.02 vs 5.09 ± 3.86, *P* < 0.001), creatinine (107.59 vs. 69.19, *P* < 0.001), creatine kinase (123.05 vs 64.80, *P* < 0.001), myoglobin (85.90 vs. 44.30, *P* < 0.001), C-reactive protein (136.00 vs 0.00, *P* < 0.001), hematocrit (39.60 ± 6.21 vs 38.77 ± 9.32, *P* < 0.001), and glucose (9.87 ± 4.69 vs 7.90 ± 5.74, *P* < 0.001). However, the triglyceride level, high-density lipoprotein, age, gender, and history of diabetes mellitus were not significantly different between two groups (Table 2).

### 3.3. Correlation between AP Severity and Serum HCY Level

Importantly, we found that the HCY levels in AP patients presenting with MOF were significantly higher than those of AP patients without MOF (14.7 vs 12.8, *P* < 0.001) (Figure 2(b)). Our Pearson correlation analysis of the APACHE scores and serum HCY levels showed that an increase of APACHE II score was significantly correlated with an increase in serum HCY level among all patients. The Pearson correlation coefficient was calculated to be 0.420 (*P* < 0.001), indicative of a positive correlation between the APACHE II score and HCY level in AP patients (Figure 2(c)).

### 3.4. ROC Analysis of HCY Level and CRP and WBC Counts

**Figure 4:**
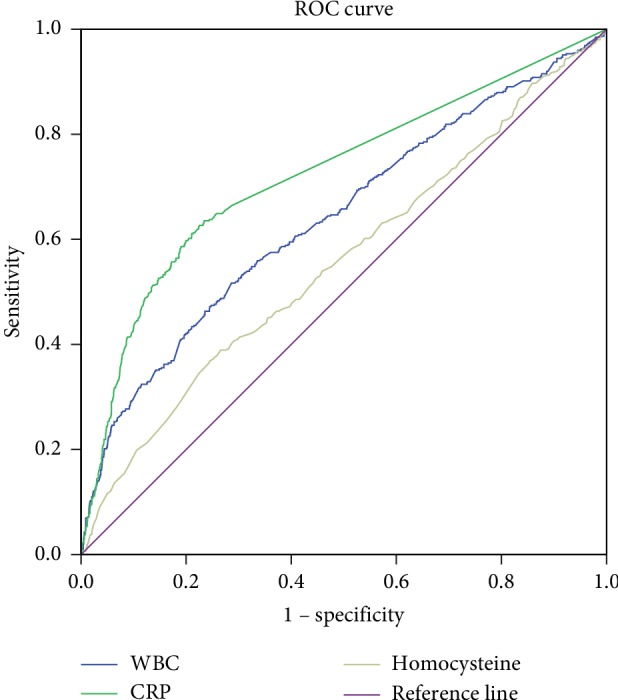
Receiver operating characteristic curve of HCY level, CRP and WBC count.

Moreover, ROC analysis shows that area under curve (AUC) of homocysteine was 0.558 (95% CI 0.526–0.589) with a sensitivity of 0.338 and a specificity of 0.789 when cutoff value was 15.75 *µ*mol/L. CRP and WBC counts for predicting multiorgan failure were 0.641 (95% CI 0.610–0.672) and 0.724 (95% CI 0.695–0.753), respectively (Figure 4). When the cutoff value was 14.395 103/*µ*L, the AUC of WBC was 0.641 (95% CI 0.610–0.672) with a sensitivity of 45.9% and a specificity of 76.4%. Moreover, the AUC of CRP was calculated to be 0.724 (95% CI 0.695–0.753) with a sensitivity of 63.1% and a specificity of 76.6% when the cutoff value was 25.5 mg/L.

### 3.5. Univariate and Multivariate Logistic Regression Analysis

In order to elucidate whether a high serum HCY level could be a risk factor for multiple organ failure, the univariate analysis was performed subsequently. The results of univariate logistic regression analysis based on these clinical risk factors including age, sex, hypertension, intensive care unit needs, APACHE II score, HCY, C-reactive protein, and etiology associated with MOF are listed in Tables 3 and 4. We found that the HCY level (odds ratio, 1.27; 95% CI, 1.121–1.460; *P* < 0.001) was a risk factor for MOF in AP patients in the univariate analysis model (Table 3). Moreover, our multivariate logistic regression analysis model showed that HCY level (hazard ratio, 1.103; 95% CI, 1.010–1.189; *P*=0.012), pancreatic necrosis (hazard ratio, 488.245; 95% CI, 261.009–913.315; *P* < 0.001), APACHE II score (hazard ratio, 1.234; 95% CI, 1.171–1.299; *P* < 0.001), ICU admission (hazard ratio, 62.018; 95% CI, 17.604–218.489; *P* < 0.001), WBC (hazard ratio, 1.058; 95% CI, 1.024–1.094; *P*=0.001), and CRP (1.008; 95% CI, 1.006–1.010, *P* < 0.001) were independently associated with MOF (Table 4).

## 4. Discussion

The relationship between multiple persistent organ failure development and the serum hyperhomocysteine (HCY) level in acute pancreatitis patients is still unknown. In this large cohort study, we unveiled that a high level of HCY might be an independent risk factor for MOF in patients with AP.

HCY is a nonessential sulfur-containing amino acid which plays key roles in the HCY-methionine cycle through interacting with vitamin B12 and folic acid [13]. HCY could also lead to the increase of oxidative stresses and was substantially involved in the formation of inflammation [14]. In the present study, we observed that AP patients in the hyperhomocysteinemia group possess significantly higher C-reactive protein levels than the control group. Based on the previous investigations, it is reasonable to presume that two potential mechanisms might be involved in such phenomena, including the induction of nitric oxide synthesis dysfunction and hyperhomocysteinemia caused by methionine diet, which could elevate thromboxane A2 activity in vessels and platelets, eventually resulting in inflammation development [14].

The development of MOF in pancreatitis patients was also known to be closely related with thrombosis and the inflammatory response. Previous reports demonstrated that early proinflammatory reactions in severe acute pancreatitis patients could lead to the development of systemic inflammatory response syndrome [15]. Cholelithiasis and biliary tract infections are the most common causes of acute pancreatitis in China. The Chinese Medical Association conducted a clinical retrospective study of the etiology of more than 6000 patients with AP in 12 hospitals across the country, biliary pancreatitis ranked first, accounting for 54.4% [16]. In this study, all HCY sample were collected within 48 h after disease onset. There are 117 alcoholic pancreatitis patients in present study, and we compared the HCY levels between alcoholic and nonalcoholic pancreatitis patients (14.78 ± 9.78 vs 13.14 ± 9.82, *P*=0.848) and found no significant differences between them. Multivariate logistic regression analysis showed that the risk of MOF in AP patients increased along with higher HCY levels, and the subsequent analysis also exhibited that the MOF ratio of the hyperhomocysteinemia group was also remarkably higher than the control group. In clinical management, the APACHE II score is one of the most commonly applied indicators for predicting the severity and mortality of pancreatitis patients. We found in this study that the APACHE II score in the hyperhomocysteinemia group was greatly higher than the control group. Moreover, our subsequent linear correlation analysis disclosed a significantly positive correlation between the HCY level and APACHE II score. But, the ROC curve based on HCY levels showed poor predictive effects of HCY levels, which is not an ideal indicator of multiple organ failure. This correlation, to a certain degree, indicated that the HCY level could be used to preliminarily evaluate the severity of AP. This also suggests that a high HCY level may predict the risk of MOF in AP patients. As we know, C-reactive protein (CRP) exerts essential roles in the development of MOF and has been previously proved to be a key risk factor for MOF. In this study, the multivariate logistic regression analysis showed that the risk ratio of C-reactive protein was 1.008 and risk ratio of HCY was 1.103. These results suggested that high HCY level, similar to CRP, could be another important risk factor for development of MOF in AP patients and could also be applied for predicting pancreatitis severity in clinics [17–19]. However, the ROC analysis showed that the performances of CRP and WBC were better than those of HCY. We think that the clinical predictive value of HCY might be lowered by the influences of diets, genetics, and other diseases.

## 5. Limitations

First of all, since only one blood sample was collected from each patient, we have no information about the changes of serum homocysteine along with the disease course. Secondly, although we analyzed clinical data of comorbidities as much as possible, the nutritional factors, environmental, or medical conditions still have potential influences on HCY level. Moreover, hyperhomocysteinemia is common in many patients and might be associated with inflammatory response. Our study here could not prove the casual relationship between HCY and MOF but suggests that HCY might be a high risk factor for MOF in pancreatitis patients, which could serve as a basis for future investigations.

## 6. Conclusion

In summary, the serum HCY level was closely correlated with MOF caused by AP. High HCY level might be an important risk factor or predictor for MOF development in AP patients.

## Figures and Tables

**Table 1:** Comparison of clinical characteristics and outcomes between the hyperhomocysteinemia group and the control group.

Variable	Control group Homocysteine level <15 *µ*mol/L *N* = 1371	Study group Homocysteine level ≥15 *µ*mol *N* = 509	*P* value
*Demographics and comorbidities*			
Median age, years, mean (SD)	49.15 (13.51)	50.93 (15.58)	0.015
Male sex, *N* (%)	751 (54.77)	372 (73.08)	<0.001
Hypertension, *N* (%)	244 (17.79)	111 (21.81)	0.039
Diabetes mellitus, *N* (%)	137 (9.99)	81 (15.91)	0.002
Apoplexy, *N* (%)	32 (2.33)	70 (13.75)	<0.001
Hyperlipidemia, *N* (%)	190 (13.86)	77 (15.13)	0.489

*Etiology*			<0.001
Biliary, *N* (%)	576 (42.01)	238 (46.76)	
Hypertriglyceridemia, *N* (%)	622 (45.37)	197 (38.70)	
Alcohol, *N* (%)	76 (5.54)	41 (8.06)	
Others, *N* (%)	97 (7.08)	33 (6.48)	

*Outcomes*			
Surgery accepted, *N* (%)	143 (10.43)	68 (13.36)	0.070
Pancreatic necrosis, *N* (%)	257 (18.7)	145 (28.5)	<0.001
ICU admission, *N* (%)	157 (11.45)	105 (20.63)	<0.001
Median hospital days (IQR)	10.00 (7.00–15.00)	10.00 (6.00–16.00)	0.576
APACHE II score, mean (SD)	5.13 (3.34)	8.46 (6.00)	<0.001
CCI, mean (SD)	1.45 (0.98)	1.69 (1.24)	0.023
Infection, *N* (%)	72 (5.25)	36 (7.07)	0.132
Renal failure and CRRT, *N* (%)	15 (1.10)	44 (8.64)	<0.001
Multiple organ failure, *N* (%)	285 (20.77)	162 (31.83)	<0.001
Mortality	28 (2.0%)	33 (6.5%)	<0.001

ICU: intensive care unit; APACHE II: acute physiology and chronic health evaluation scoring system II; SD: standard deviation; IQR: interquartile range; CRRT: continuous renal replacement therapy; CCI: Charlson Comorbidity Index.

**Table 2:** Comparison of variables between the MOF group and the non-MOF group.

Variable	Non-MOF group *N* = 1433	MOF group *N* = 447	*P* value
*Demographics and comorbidities*			
Median ages, years, mean (SD)	51.14 (14.24)	49.16 (14.05)	0.826
Male sex, *N* (%)	869 (60.64)	254 (56.82)	0.151
Hypertension, *N* (%)	253 (17.66)	102 (22.82)	0.030
Diabetes mellitus, *N* (%)	203 (14.17)	66 (14.77)	0.916
Apoplexy, *N* (%)	81 (5.65)	21 (4.70)	0.109
Hyperlipidemia, *N* (%)	200 (13.96)	67 (14.98)	0.800

*Etiology*			0.002
Biliary, *N* (%)	595 (41.52)	219 (49.00)	
Hypertriglyceridemia, *N* (%)	633 (44.17)	186 (41.61)	
Alcohol, *N* (%)	91 (63.50)	26 (5.82)	
Others, *N* (%)	114 (7.96)	16 (3.58)	

*Outcomes*			
Surgery accepted, *N* (%)	112 (7.82)	99 (22.15)	<0.001
Pancreatic necrosis, *N* (%)	23 (1.61)	379 (84.79)	<0.001
ICU admission, *N* (%)	8 (0.56)	254 (56.82)	<0.001
Median hospital days (IQR)	9.00 (6.00–13.00)	16.00 (10.00–27.00)	<0.001
APACHE II score, mean (SD)	4.22 (2.47)	9.81 (5.36)	<0.001
CCI, mean (SD)	1.37 (0.88)	2.30 (1.67)	<0.001
Infection, *N* (%)	2 (0.14)	106 (23.71)	<0.001
Mortality, *N* (%)	0 (0)	61 (13.65)	<0.001
CRP, mg/L (IQR)	0 (0.00–14.00)	136 (0.00–219.00)	<0.001
WBC, ×10^3^/*µ*L, mean (SD)	14.78 ± 6.53	11.76 ± 5.65	<0.001
PCT, (IQR)	0.00 (0.00–0.16)	0.00 (0.00–0.55)	0.287
HCY, *µ*mol/L (SD)	12.78 (9.53)	14.69 (10.59)	<0.001
HCT, %, mean (SD)	38.77 (9.32)	39.60 (6.21)	<0.001
BUN, mmol/L, mean (SD)	5.09 (3.86)	8.17 (7.02)	<0.001
Creatinine, *µ*mol/L, mean (SD)	69.19 (58.69)	107.59 (106.06)	<0.001
TG, mmol/L, mean (SD)	4.61 (7.82)	4.59 (5.61)	0.789
HDL-C, mmol/L, mean (SD)	0.96 (0.54)	0.78 (0.55)	0.254
LDL-C, mmol/L, mean (SD)	2.41 (1.71)	2.44 (1.29)	0.716
LDH, U/L (IQR)	226.60 (169.80–316.33)	462.82 (285.90–743.05)	<0.001
Glucose, mmol/L, mean (SD)	7.90 (5.74)	9.87 (4.69)	0.014
TC, mmol/L, mean (SD)	5.53 (4.53)	5.04 (4.47)	0.086
TBA, *µ*mol/L (IQR)	2.10 (0.80–5.30)	2.80 (1.20–7.40)	0.273
Myoglobin, *µ*g/L (IQR)	44.30 (31.30–65.60)	85.90 (48.60–225.05)	<0.001
Creatine kinase, U/L (IQR)	64.80 (41.40–108.10)	123.05 (50.33–369.40)	<0.001

WBC: white blood cell; HCT: hematocrit; PCT: procalcitonin; CRP: C-reactive protein; BUN: blood urea nitrogen; TC: total cholesterol; TG: triglyceride; HDL-C: high-density lipoprotein cholesterol; LDL-C: low-density lipoprotein cholesterol; LDH: lactic dehydrogenase; TBA: total bile acids.

**Table 3:** Univariate logistic regression analysis of multiple organ failure in AP patients.

Variable	Regression coefficient B	OR (95% CI)	*P* value
Age	0.010	1.010 (1.002–1.017)	0.10
Male sex	0.158	1.171 (0.944–1.452)	0.151
Hypertension	0.287	1.332 (1.028–1.727)	0.030
Diabetes mellitus	0.016	1.016 (0.752–1.373)	0.916
APACHE II score	0.536	1.709 (1.614–1.809)	<0.001
CCI	0.085	1.089 (0.912–1.136)	0.059
Homocysteine	0.232	1.271 (1.121–1.460)	<0.001
Pancreatic necrosis	5.834	341.682 (210.124–555.607)	<0.001
Surgery accepted	1.211	3.355 (2.498–4.507)	<0.001
ICU admission	5.457	234.424 (114.145–481.442)	<0.001
WBC	0.088	1.092 (1.073–1.113)	<0.001
CRP	0.008	1.008 (1.007–1.009)	<0.001

*Etiology (reference to others)*			
Biliary pancreatitis	0.964	2.622 (1.520–4.526)	0.001
Hypertriglyceridemia	0.739	2.094 (1.210–3.622)	0.008
Alcohol	0.711	2.036 (1.030–4.022)	0.041
HCT	−0.017	0.984 (0.969–0.998)	0.030
BUN	0.128	1.137 (1.107–1.167)	<0.001
Creatinine	0.007	1.007 (1.005–1.008)	<0.001
Glucose	0.081	1.084 (1.057–1.112)	<0.001
TG	0.000	1.000 (0.985–1.014)	0.952
HDL	−1.005	0.366 (0.272–0.493)	<0.001
LDH	0.004	1.004 (1.003–1.004)	<0.001
Myoglobin	0.004	1.004 (1.003–1.005)	<0.001
Creatine kinase	0.001	1.001 (1.001–1.002)	<0.001

**Table 4:** Multivariate logistic regression analysis of multiple organ failure in AP patients.

Variable	Regression coefficient B	OR (95% CI)	*P* value
Age	0.008	1.008 (0.988–1.027)	0.435
Male sex	0.360	1.427 (0.839–2.426)	0.189
Hypertension	−0.152	0.845 (0.444–1.609)	0.608
Pancreatic necrosis	5.214	488.245 (261.009–913.315)	<0.001
APACHE II score	0.229	1.234 (1.171–1.299)	<0.001
Surgery accepted	0.765	2.15 (0.870–5.309)	0.097
ICU admission	4.127	62.018 (17.604–218.489)	<0.001

*Etiology (reference to others)*			
Biliary pancreatitis	0.865	2.406 (0.808–7.171)	0.115
Hypertriglyceridemia	0.450	1.758 (0.589–5.251)	0.312
Alcohol	−0.582	0.419 (0.095–1.846)	0.250
WBC	0.057	1.058 (1.024–1.094)	0.001
CRP	0.008	1.008 (1.006–1.010)	<0.001
BUN	0.038	1.040 (0.983–1.101)	0.174
HDL	−0.225	0.778 (0.392–1.545)	0.473
Homocysteine	0.101	1.103 (1.010–1.189)	0.012

## Data Availability

The data used to support the findings of this study are available upon request to the corresponding author.

## Abbreviations

HCY: Homocysteine
APACHE II: Acute physiology and chronic health evaluation II
ICU: Intensive care unit
AP: Acute pancreatitis
SAP: Severe acute pancreatitis
SIRS: Systemic inflammatory response syndrome
IQR: Interquartile range
MOF: Multiple organ failure
CRP: C-reactive protein
HCT: Hematocrit
BUN: Blood urea nitrogen
LDH: Lactate dehydrogenase
PCT: Procalcitonin
TC: Total cholesterol
TBA: Total bile acid
HDL-C: High-density lipoprotein cholesterol
LDL-C: Low-density lipoprotein cholesterol
APACHE: The Acute Physiology and Chronic Health Evaluation (APACHE)
